# Plasma Chemokine CCL2 and Its Receptor CCR2 Concentrations as Diagnostic Biomarkers for Breast Cancer Patients

**DOI:** 10.1155/2018/2124390

**Published:** 2018-07-30

**Authors:** Emilia Lubowicka, Andrzej Przylipiak, Monika Zajkowska, Barbara Maria Piskór, Paweł Malinowski, Wojciech Fiedorowicz, Sławomir Ławicki

**Affiliations:** ^1^Department of Esthetic Medicine, Medical University of Bialystok, 15-267 Bialystok, Poland; ^2^Department of Biochemical Diagnostics, Medical University of Bialystok, 15-269 Bialystok, Poland; ^3^Department of Oncological Surgery, Bialystok Oncology Center, Bialystok, 15-276 Bialystok, Poland

## Abstract

The aim of this study was to investigate plasma levels and applicability of CCL2, CCR2, and tumor marker CA 15-3 in breast cancer (BC) patients and in relation to the control groups: patients with benign breast tumor and healthy subjects. Plasma levels of tested parameters were determined by enzyme-linked immunosorbent assay (ELISA) and CA 15-3 by Chemiluminescent Microparticle Immunoassay (CMIA). The median levels of CCL2 in entire group of BC were significantly higher compared to the control groups, similarly as median levels of CA 15-3. CCR2 is a negative marker whose levels were significantly lower in BC group compared to healthy women. The concentration of CCL2 in BC increases with advancing tumor stage, while a median level of CCR2 decreases with advancing stage. CCL2 showed the highest value of sensitivity (SE) (64.95%) in entire BC group and also in early stages of disease. The highest specificity (SP) was obtained by CA 15-3 (85.71%). The area under the ROC curve (AUC) of CCR2 (0.7304) was the largest of all the tested parameters (slightly lower than CA 15-3) in the entire BC group, but a maximum range was obtained for the combination of all tested parameters with CA 15-3 (0.8271). In early stages of BC the highest AUC of all tested parameters was observed in CCL2 or CCR2 (stage I: 0.6604 and 0.6564; respectively; stage II: 0.7768, respectively, for CCR2). The findings of this study suggest that there may be applicability of CCL2, CCR2 in diagnosis of BC patients, particularly in conjunction with CA 15-3.

## 1. Introduction

Breast cancer is one of the most frequent malignancies in women around the world, with a higher incidence in developed countries and greatest relative mortality in less developed countries [1, 2]. Tumor growth and metastasis are regulated at least partially by chemokine-chemokine receptor interactions. The family currently includes more than 50 members of the human chemokines and the corresponding 20 chemokine receptors and they are classified into several groups depending on composition of a conserved cysteine motif present on the ligand [3, 4].

Chemokines are a family of small soluble proteins involved not only in inflammation but also in important physiological and pathological processes, such as cancer progression and metastasis [5]. Carcinogenesis is thought to intensify by prolonged inflammation providing a microenvironment that is ideal for cancer development and growth [6]. Many cancer cells, including breast cancer cells, express chemokines and chemokine receptors [7].

Among more than 50 human chemokines, CC-chemokine ligand 2 (CCL2) is of particular importance. CCL2, also known as monocyte chemotactic protein-1 (MCP-1), belongs to the CC family of chemokines. CCL2 is produced by variety of cell types, not only by tumor cells but also by stromal cells such as monocytes, fibroblasts, and endothelial cells [8]. The activity of CCL2 is mediated through its binding to the receptor CCR2 [9]. CCR2 is a receptor that binds other chemokines, particularly CCL8, CCL7, and CCL13 consistent with their structural similarity to CCL2. This flexibility in chemokine-receptor interaction may lead to different biological functions, depending on the particular chemokine and receptor pair engaged, or may produce similar effects, suggesting that most of chemokines have redundant or similar functions with other known chemokines [10]. Both CCL2 and its receptor CCR2 have been detected in most tumors, including those of the breast, endometrium, colon, and prostate [11–15].

The aim of the current study was to determine the plasma levels of CCL2, CCR2 and the levels of the commonly accepted tumor marker (CA 15-3) in 3 groups: (1) the breast cancer patients group; (2) the benign breast tumor group; and (3) the control group consisting of healthy women. We evaluated the plasma levels of these markers in different stages of breast cancer. Additionally, we defined the criteria for the diagnosis based on investigated marker set. Obtained data may be helpful in both determining the clinical applicability of the analyzed parameters (separately and in conjunction) in the diagnosis of breast cancer and in the differentiation of its subtypes.

## 2. Material and Methods

### 2.1. Human Subjects

Table 1 shows the examined and control groups. The study comprised 100 patients with breast cancer (BC) who were referred to the Department of Oncology, Medical University of Bialystok, Poland, between 2015 and 2017. Tumor classification and staging were determined in accordance with the International Union against Cancer Tumor-Node-Metastasis (UICC-TNM) classification in all cases. Breast cancer histopathology was established in all cases by tissue biopsy of the mammary tumor or after surgery from tumor cancer tissues (all patients with* ductal adenocarcinoma)*. Written consent including participants' own statements regarding their medical history (i.e., data related to reproductive history, personal or family history of cancer, general health issues: hospitalization or surgery, and use of medications) and lifestyle habits including smoking was obtained from all the subjects. None of the patients had received chemo- or radiotherapy before blood sample collection. The pretreatment staging procedures included physical and blood examinations, mammography, mammary ultrasound scanning, breast core biopsies, and chest X-rays. In addition, radioisotopic bone scans, the examination of bone marrow aspirates, and CT scans of the brain and chest were performed where necessary.

The control groups included 35 patients with benign breast tumors (*adenoma, fibroadenoma*) and 35 healthy, untreated women who underwent mammary gland examination performed by a gynecologist prior to blood sample collection. In addition, mammary ultrasound scanning was performed in all cases. Benign breast tumor histopathology was established in all cases by tissue biopsy of the mammary tumor or after surgery.

We have selected a control group in the best way and exclude women with other diseases that could influence the quality of our research. From the control group we had to exclude people with inflammatory conditions, cardiovascular disorders, and other accompanying diseases. For this reason, the number of patients with benign breast tumor was chosen according to the number of healthy subjects.

The study was approved by the local Ethics Committee (R-I-002/51/2015) and all the patients gave their informed consent for participation in the study.

### 2.2. Plasma Collection and Storage

Venous blood samples were collected from each patient. Blood was collected into EDTA tubes (S-Monovette, SARSTEDT, Germany), centrifuged 1000 x g for 15 min at 2-8°C to obtain plasma samples, and stored at −85°C until assayed.

### 2.3. Measurement of CCL2, CCR2, and CA 15-3

The tested parameters (CCL2 and CCR2) were measured with enzyme-linked immunosorbent assay (ELISA) (CCL2-R&D systems, Abingdon, United Kingdom; CCR2-EIAab Science, Wuhan, China), according to the manufacturer's instructions. ELISA system can specifically and selectively detect soluble chemokine receptors [16–18]. Plasma concentration of CA 15-3 was measured by chemiluminescent microparticle immunoassay (CMIA) (Abbott, Chicago, IL, USA). The intra-assay coefficient of variation (CV%) of CA 15-3 is reported to be 2.2% at a mean concentration of 27.0 U/mL, SD=0.6. CCL2 is reported to be 4.7% at a mean concentration of 364 pg/mL, SD=17.1 and CCR2 is reported to be ≤ 7.3%. The inter-assay coefficient of variation (CV%) of CA 15-3 is reported to be 2.6% at a mean concentration of 27.0 U/ml, SD=0.7. CCL2 is reported to be 5.8% at a mean concentration of 352 pg/mL, SD=20.5 and CCR2 to be ≤10.9%. The value of intra- and interassay CVs were calculated by the manufacturers and enclosed in the reagent kits. The assay does not exhibit cross-reactivity or interference with numerous human cytokines and other growth factors. Duplicate samples were assessed for each patient.

### 2.4. Statistical Analysis

Statistical analysis was performed using STATISTICA 12.0 (StatSoft, Tulsa, OK, USA). Preliminary statistical analysis (using the Shapiro-Wilk test) revealed that the tested parameters and tumor marker levels did not follow a normal distribution. Consequently, the statistical analysis between the groups was performed by using the Mann–Whitney U test, the Kruskal–Wallis test, and a multivariate analysis of various data by the post hoc Dwass–Steel–Critchlow–Fligner test. Statistically significant differences were defined as comparisons resulting in p<0.05. Diagnostic sensitivity (SE) and specificity (SP) were calculated. The* cut-off* values were calculated by Youden's index (as a criterion for selecting the optimum cut-off point) and each of the tested parameters was as follows: CCL2: 183.19 pg/mL; CCR2: 1.49 ng/mL; and CA 15-3: 16.85 U/mL. In the analyses of both diagnostic performance (SE, SP) and ROC curve, healthy subjects and benign breast tumor group were used as a control group. The construction of the ROC curves was performed using the GraphRoc program for Windows (Windows, Royal, AR, USA) and the areas under the ROC curve (AUC) were calculated to evaluate the diagnostic accuracy and to compare AUC for all tested parameters separately and in combination with the commonly used tumor marker (CA 15-3).

## 3. Results

Table 2 presents the median and the range of plasma levels of the investigated parameters and CA 15-3 in examined groups. The median levels of CCL2 and CA 15-3 in the entire group of BC were significantly higher than in the healthy patients group (p<0.05 in all cases). Moreover, the median levels of CCR2 in the group of BC were significantly lower when compared to the healthy subjects (p<0.001). We also noticed that the median of CCL2 levels in stages III and IV was significantly higher when compared to healthy group (p<0.001). In the case of CCR2, the median of plasma levels in all stages of BC was significantly lower when compared to healthy subjects (p<0.001 in all cases). However, the concentrations of commonly accepted tumor marker (CA 15-3) in all stages of BC were significantly higher than in the healthy volunteers.

The statistical test also showed the similar relationship between the entire group of BC and patients with benign breast tumors. The concentrations of CCL2 and CA 15-3 in the group of BC were significantly higher than in the benign breast tumors (p<0.001). We also observed that the median of CCL2 and commonly accepted tumor marker (CA 15-3) levels in all stages of BC were significantly higher when compared to benign breast tumors. However, the concentration of CCR2 in stages II, III, and IV of BC was significantly lower when compared to the patients with benign breast tumors.

The CCL2 ELISA data showed that plasma levels of CCL2 were significantly increased in the entire BC group compared to the total control group (benign breast tumor and healthy subjects) (p<0.001), similarly as the median levels of CA 15-3 (p<0.001). However, the concentration of CCR2 in patients with breast cancer was significantly lower than entire control group (p<0.001). The same relationships were observed in all stages of BC (p<0.05 in all cases).

Furthermore, the concentration of CCL2 in BC group increases with advancing tumor stage. We detected significantly higher plasma levels of CCL2 and commonly accepted tumor marker CA 15-3 in the comparison of stages III and IV to stage I (*p*<0.001) and to stage II (*p*=0.001).

We also noticed statistical differences between the concentrations of CCL2 and CCR2 in patients with benign breast tumors and healthy controls (p<0.05). However, CA 15-3 did not show statistical difference in plasma levels between two studied control groups. This demonstrates possible chance of using CCL2 and CCR2 in differentiation between benign breast tumor and healthy women.

Table 3 shows the diagnostic criteria: sensitivity (SE), specificity (SP), predictive value of a positive test result (PPV), and predictive value of a negative test result (NPV) in breast cancer patients. The sensitivity of the tested parameters in the total cancer group was higher for CCL2 (64.95%) than for CCR2 (61.86%) and higher than for routinely used tumor marker CA 15-3 (59.79%). A maximum diagnostic sensitivity (90.72%) was obtained for the combination of CCL2 and CCR2 with CA 15-3. Among all the parameters, the highest SE in early stages of cancer was observed for CCL2 (in stage I of BC: 58.82%, in stage II: 58.54%). The combined use of the tested parameters with CA 15-3 resulted in an increase of SE in every stage of BC. The diagnostic SP of the tested parameters was the highest for CA 15-3 (85.71%) in the entire group of breast cancer patients. Moreover, in all stages of cancer, CA 15-3 has the highest specificity (85.71% in all cases).

Among the examined parameters, the predictive value of a positive test result in the group of BC patients was the highest for CCL2 (78.75%) in comparison to CCR2 (77.92%) but was lower than PPV for CA 15-3 (86.57%). The highest PPV values in all stages of cancer were observed for CA 15-3 (65.38%, 70.97%, and 67.86%, respectively). The predictive value of a negative test result in the group of BC was higher for CCL2 (57.50%) than for CCR2 (55.42%) and was slightly lower than NPV for CA 15-3 (58.06%). The combination of CCL2 or CCR2 with antigen CA 15-3 resulted in an increase in the NPV. The highest NPV values in other stages of BC (II, III, and IV) were observed for CA 15-3 (70.97% and 94.74%, respectively). The combination of CCL2 or CCR2 with antigen CA 15-3 resulted in an increase in the NPV in all stages of cancer.

**Figure 1:**
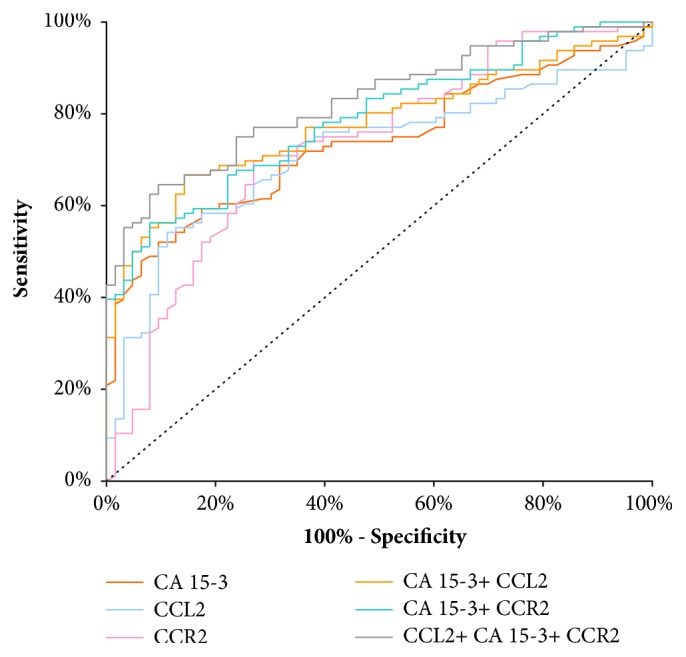
Diagnostic criteria of ROC curve for tested parameters and in combination with CA 15-3 in entire BC group.

The relationship between the diagnostic SE and SP is illustrated by the ROC curve (Table 4). The AUC indicates the possible clinical usefulness of a tumor marker and its diagnostic power. We have noticed that the AUC for CA 15-3 (0.7354) in entire group of BC was slightly larger than for CCR2 (0.7304) and CCL2 (0.7154). Moreover, areas under the ROC curve for all parameters were significantly larger in comparison to AUC=0.5 (borderline of the diagnostic usefulness of the test) (*p*<0.001 in all cases). The combination of CCL2 or CCR2 with CA 15-3 resulted in an increase in areas under the ROC curve in all cases (0.7771 and 0.7879; respectively). A maximum range in the entire BC group was obtained for the combination of all the studies parameters (0.8271; p<0.001) (Figure 1).

**Figure 2:**
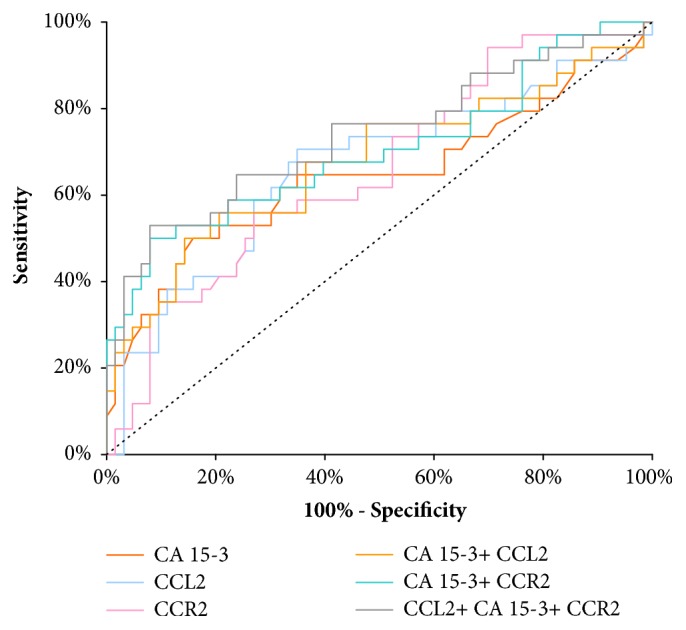
Diagnostic criteria of ROC curve for tested parameters and in combination with CA 15-3 in stage I of BC.

**Figure 3:**
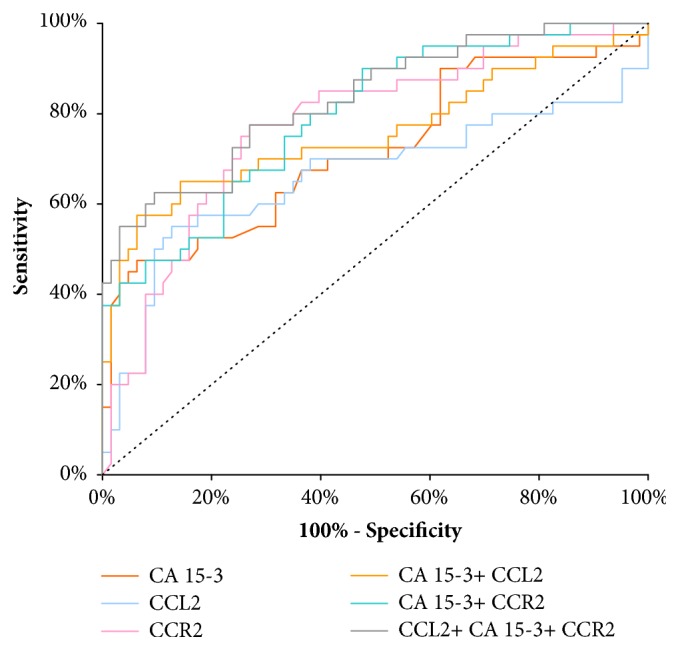
Diagnostic criteria of ROC curve for tested parameters and in combination with CA 15-3 in stage II of BC.

**Figure 4:**
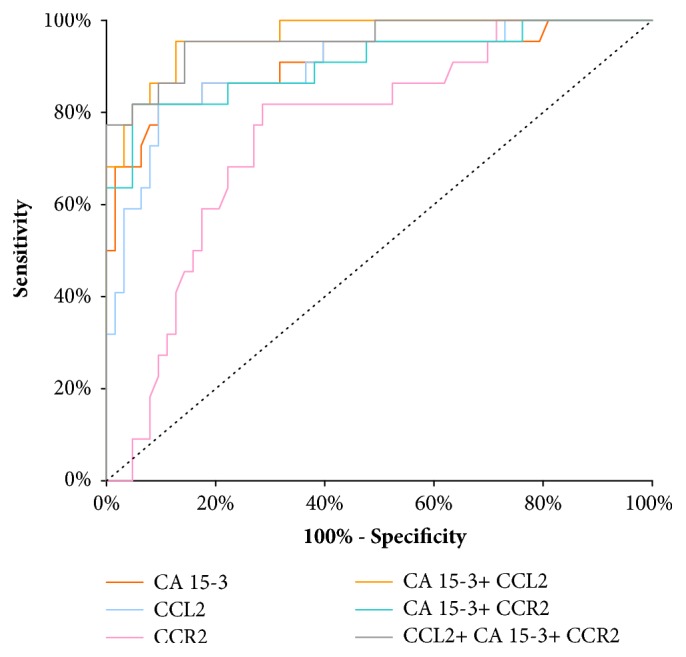
Diagnostic criteria of ROC curve for tested parameters and in combination with CA 15-3 in stages III and IV of BC.

The AUC of CCL2 presented noticeable increase with the BC stage advancement, in parallel to CA 15-3 AUC. In stage I of BC the highest AUC of all the tested parameters was presented by CCL2 (0.6604) and it was the parameter which was significantly larger in comparison to AUC= 0.5 (p=0.0097), correspondingly to CCR2 (p=0.0074) and CA 15-3 (p=0.0266) (Figure 2). In stage II of BC the highest AUC of all tested parameters was observed in CCR2 (0.7768; p<0.001); importantly it was higher than that of CA 15-3 (0.7163). Moreover, the AUCs for CCL2 and CCR2, CA 15-3, were significantly higher in comparison to AUC =0.5 (p=0.0097; p=0.0074; p=0.0266; respectively) (Figure 3). In stages III and IV of BC the highest AUC from all the tested parameters was observed for CA 15-3 (0.9098; p<0.001) and of note, it was slightly higher than for CCR2 (0.8983; p< 0.001). Additionally, the AUCs for CCL2 and CCR2, CA 15-3, were significantly larger in comparison to AUC =0.5 (p<0.001 in all cases). The combination of CCL2 or CCR2 with antigen CA 15-3 resulted in an increase in the AUCs in all cases (Figure 4).

## 4. Discussion

Recent studies have showed that chemokines family and their receptors play crucial roles in the development of breast cancer, including tumor growth, migration, and angiogenesis [19–21]. Moreover, they influence the infiltration of leukocytes into any tissue, including tumors [22]. Out of all the known chemokines, breast cancer cells express the CCL2 chemokine, the receptor of which is CCR2 [23]. CCL2 may promote breast cancer cell development by many mechanisms. CCL2 mainly binds to CCR2 receptor, but other receptors including CCR4 can also be involved in activation of signaling pathways [20]. As an important component in the tumor microenvironment, leukocytes in cancer stroma support tumor growth and facilitate metastatic dissemination [24]. CCL2 recruits CCR2-expressing inflammatory monocytes to facilitate breast tumor metastasis [25]. However, CCL2-CCR4 signaling also regulates the migration and infiltration of T regulatory cells to tumor sites [26].

CCL2 (MCP-1) is proinflammatory chemokine; therefore, studies have demonstrated its overexpression or increased serum levels and resultant promotion of tumor growth in breast [27–29]. The data presented here reveals that breast cancer patients have a significantly higher level of plasma CCL2 and commonly used tumor marker CA 15-3 than control groups. Moreover, the concentration of CCR2 in the group of BC was significantly lower when compared to the healthy subjects. There have been studies showing that MCP-1 median (range) serum level was markedly elevated in cancer patients versus controls [30]. In the study by Dwyer et al. [31] the ELISA method was used to detect MCP-1 serum levels in 125 BC patients and 86 age-matched controls. Results of the study revealed that BC patients showed higher levels of MCP-1, but the difference was not statistically significant. One of the findings in our study was that of a statistically significantly increase of plasma CCL2 concentration in breast cancer patients compared to benign breast tumors. We are able to establish CCL2 as differentiation maker between malignant and benign breast diseases. Additionally, CCL2 and CCR2 levels may be useful in differentiation between benign breast tumor and healthy women. The work of Lebrecht and her coworkers [11] in the 2004 show the analysis of serum MCP-1 level in patients with invasive breast cancer, ductal carcinoma* in situ*, benign breast lesions, and healthy women. The authors failed to show any significant differences in the serum chemokine levels of these four groups.

However, the limitations of this study (smaller number of patients in control groups than patients with breast cancer) make it important to confirm our results on a larger group of these patients but the numbers of healthy subjects and patients with benign breast cancer are large enough for regular statistic evaluation.

Interestingly, we observed significant increase of CCL2 serum levels in cancer patients based on stage of disease compared with entire control group. In some studies, elevated expression or serum levels of CCL2 have been strongly associated with advanced stage of disease in BC [30, 32–34]. There is some evidence to suggest that high levels of tumor-associated macrophages (TAMs) correlate with poor prognosis in breast cancer, and it has been suggested that the relationship between high levels of CCL2 chemokine and poor prognosis is mediated by the recruitment of TAMs into the tumor microenvironment [25, 35]. TAMs contribute to tumor progression also by producing chemokine CCL2, which leads to the elevated angiogenic profile attributed to this chemokine [36, 37].

In the present study, we defined the diagnostic criteria (sensitivity and specificity) of all tested parameters. The ability of a test to correctly classify an individual as diseased is called the diagnostic sensitivity. In our study, the sensitivity of CCL2 was the highest of all the tested parameters (64.95%) in entire BC group. Additionally, a maximum diagnostic sensitivity (90.72%) was obtained for the combination of CCL2 and CCR2 with CA 15-3. Specificity is the ability of a test to correctly classify an individual as disease-free. Our results showed that the diagnostic specificity was the highest for CA 15-3 (85.71%). According to our knowledge, there are no studies of the diagnostic criteria (sensitivity and specificity) of CCL2 and CCR2 serum or plasma levels in breast cancer patients. The results of the study on the role of CCL2 in various cancers indicated that specificity and sensitivity were 80.00% and 85.00% for CCL2 to differentiate CNS (central nervous system) tumor patients from nontumoral individuals [38]. In our previous study in BC [39], which comprised other cytokines (M-CSF and VEGF) in breast cancer patients, the highest SE value was found for VEGF (76.25%) and it was higher than M-CSF (60%).

The ROC curve illustrates the relationship between diagnostic sensitivity and specificity. The area under the ROC curve indicates the clinical applicability of a tumor marker. To date, there are no reports of the diagnostic usefulness of CCL2 and CCR2 serum or plasma levels in breast cancer patients. According to this study, the ROC area of CA 15-3 (0.7354) was the largest of all the tested parameters in the group of BC and slightly larger than for CCR2 (0.7304) and CCL2 (0.7154). Moreover, ROC curve analysis using the combination CCL2+CCR2+CA 15-3 (AUC = 0.8271) showed an interesting improvement in BC diagnosis as compared with CCL2 or CCR2 alone. In the paper by Koper et al. [38], the area under the ROC curve for CCL2 (AUC=0.793) in differentiation between CNS tumor patients and nontumoral individuals was slightly larger than ours [38]. The results obtained from the experiments conducted with pleural liquids showed that AUC value for CCL2 was 0.7912 to differentiate MPM (malignant pleural mesothelioma) from ADCA (adenocarcinomas) and BPE (benign pleural effusions) [40]. The right direction of the diagnostic power assessment is a combined analysis of the tested parameters, which was demonstrated also in our previous studies on other cytokines (M-CSF, VEGF) in breast cancer patients [41, 42]. In our previous study in BC [39], which comprised other cytokines (M-CSF and VEGF) in breast cancer patients, the highest AUC value was found for VEGF (0.729) and it was higher than M-CSF (0.645). We have verified that CCL2 and CCR2 may be more applicable and useful than M-CSF. However, the ROC area of VEGF was larger than CCL2, and the same relationship was observed in our previous papers [41, 43]

The complete and current state of knowledge leads to the conclusion that increased levels of chemokines in plasma may help tumor cells to migrate and invade. In this study, we are trying to draw attention to a CCL2 chemokine and its receptor CCR2, the levels of which are statistically significantly different in breast cancer patients compared to control groups. Additionally, CCL2 and CCR2 levels may be useful in differentiation between benign breast tumor and healthy women. The area under ROC curve was the highest for the combination of CCL2 and CCR2 with commonly accepted tumor marker, which indicates a possible clinical significance of plasma CCL2 and CCR2 measurements in the diagnosis of BC. The findings of this study suggest there may be applicability of CCL2 and CCR2 in diagnosis of BC patients, particularly in conjunction with CA 15-3.

## Figures and Tables

**Table 1:** Characteristics of breast cancer patients and control group.

**Study group**			**Number of patients**
*EXAMINED GROUPS*	**Breast cancer patients**	*Ductal adenocarcinoma *	100
	Median age (range)		57 (21-84)
	Tumor stage	I	34
		II	41
		III and IV	25
	Menopausal status:		
	(i) premenopausal		22
	(ii) postmenopausal		78

*CONTROL GROUPS*	**Benign breast tumor group**		35
		*adenoma*	12
		*fibroadenoma*	23
	Median age (range)		39 (21-63)
	Menopausal status:		
	(i) premenopausal		12
	(ii) postmenopausal		23
	**Healthy women**		35
	Median age (range)		37 (21-58)
	Menopausal status:		
	(i) premenopausal		15
	(ii) postmenopausal		20

**Table 2:** Plasma levels of examined parameters and CA 15-3 in patients with breast cancer and in control groups.

**Groups tested**	**CCL2**	**CCR2**	**CA 15-3**
	**(pg/mL)**	**(ng/mL)**	**(U/mL)**
Breast cancer (median, range)			

Stage I	192.81 (36.68-438.58)^**b/f**^	1.34 (0.11-8.17)^**a/f**^	17.15 (6.20-50.30)^**a/b/f**^
Stage II	227.22 (1.45-1631.48)^**b/f**^	0.93 (0.05-6.16)^**a/b/f**^	17.60 (4.40-48.10)^**a/b/f**^
Stage III and IV	398.55 (99.12-592.30)^**a/b/c/d/f**^	0.92 (0.34-3.81)^**a/b/f**^	27.75 (8.90-250.00)^**a/b/c/d/f**^
Total group	228.34 (1.45-1631.48)^**a/b/f**^	0.96 (0.05-8.17)^**a/f**^	19.20 (4.40-250.00)^**a/b/f**^

Control groups (median, range)			

Benign breast tumor	118.64 (37.37-489.88)^**e**^	1.82 (0.05-7.67)^**e**^	14.00 (5.20-20.70)
Healthy women	155.17 (70.58-440.06)	3.45 (0.84-22.60)	13.40 (6.30-28.40)
Total control group	130.64 (37.37-489.88)	2.30 (0.05-22.60)	13.60 (5.20-28.40)

**Notes:** ^a^statistically significant when patients with BC compared with healthy women.^b^Statistically significant when patients with BC compared with benign breast tumor group. ^c^Statistically significant when patients with BC stages III and IV compared with patients with BC stage I. ^d^Statistically significant when patients with BC stages III and IV compared with patients with BC stage II. ^e^Statistically significant when patients with benign breast tumor compared with healthy women. ^f^Statistically significant when patients with BC compared with total control group.

**Table 3:** Diagnostic criteria of tested parameters and in combined analysis with CA 15-3 in breast cancer patients.

**Tested parameters**	**Diagnostic criteria (**%**)**	**Breast cancer**			
		**Stage I**	**Stage II**	**Stage III/IV**	**Total group**
CCL2	SE	58.82	58.54	86.36	64.95
	SP	73.02	73.02	73.02	73.02
	PPV	54.05	58.54	52.78	78.75
	NPV	76.67	73.02	93.88	57.50

CCR2	SE	55.88	58.50	77.27	61.86
	SP	73.02	73.02	73.02	73.02
	PPV	52.78	58.54	50.00	77.92
	NPV	75.41	73.02	90.20	55.42

CA 15-3	SE	50.00	53.66	86.36	59.79
	SP	85.71	85.71	85.71	85.71
	PPV	65.38	70.97	67.86	86.57
	NPV	76.06	73.97	94.74	58.06

CCL2+ CA 15-3	SE	73.53	70.73	95.45	77.32
	SP	63.49	63.49	63.49	63.49
	PPV	52.08	55.77	47.73	76.53
	NPV	81.63	76.92	97.56	64.52

CCR2+ CA 15-3	SE	70.59	82.93	86.36	79.38
	SP	65.08	65.08	65.08	65.08
	PPV	52.17	60.71	46.34	77.78
	NPV	80.39	85.42	93.18	67.21

CCL2+CCR2+ CA 15-3	SE	85.29	92.68	95.45	90.72
	SP	44.44	44.44	44.44	44.44
	PPV	45.31	52.05	37.50	71.54
	NPV	84.85	90.32	96.55	75.68

**Table 4:** Diagnostic criteria of ROC curve for tested parameters and CA 15-3.

**Tested parameters**	**AUC**	**SE**	**95% C.I. (AUC)**	***p* (AUC=0.5)**
	*ROC criteria in breast cancer (total group)*			

CCL2	0.7154	0.0410	0.635-0.796	**<0.001**
CCR2	0.7304	0.0412	0.650-0.811	**<0.001**
CA 15-3	0.7354	0.0389	0.659-0.812	**<0.001**
CCL2+ CA 15-3	0.7771	0.0363	0.706-0.848	**<0.001**
CCR2+ CA 15-3	0.7879	0.0349	0.719-0.856	**<0.001**
CCL2+ CCR2+ CA 15-3	0.8271	0.0316	0.765-0.889	**<0.001**

	*ROC criteria in breast cancer (I stage)*			

CCL2	0.6604	0.0620	0.539-0.782	**0.0097**
CCR2	0.6564	0.0584	0.542-0.771	**0.0074**
CA 15-3	0.6452	0.0655	0.517-0.774	**0.0266**
CCL2+ CA 15-3	0.6783	0.0620	0.557-0.800	**0.0040**
CCR2+ CA 15-3	0.7031	0.0606	0.584-0.822	**<0.001**
CCL2+ CCR2+ CA 15-3	0.7367	0.0575	0.624-0.849	**<0.001**

	*ROC criteria in breast cancer (II stage)*			

CCL2	0.6615	0.0625	0.539-0.784	**0.0097**
CCR2	0.7768	0.0479	0.683-0.871	**<0.001**
CA 15-3	0.7163	0.0551	0.608-0.824	**<0.001**
CCL2+ CA 15-3	0.7575	0.0541	0.652-0.863	**<0.001**
CCR2+ CA 15-3	0.7940	0.0448	0.706-0.882	**<0.001**
CCL2+ CCR2+ CA 15-3	0.8317	0.0416	0.750-0.913	**<0.001**

	*ROC criteria in breast cancer (III and IV stages)*			

CCL2	0.8983	0.0417	0.817-0.980	**<0.001**
CCR2	0.7605	0.0570	0.649-0.872	**<0.001**
CA 15-3	0.9098	0.0426	0.826-0.993	**<0.001**
CCL2+ CA 15-3	0.9654	0.0184	0.929-1.001	**<0.001**
CCR2+ CA 15-3	0.9076	0.0437	0.822-0.993	**<0.001**
CCL2+ CCR2+ CA 15-3	0.9582	0.0248	0.909-1.007	**<0.001**

*p*: statistically significantly larger AUC compared to AUC=0.5.

## Data Availability

The data used to support the findings of this study are available from the corresponding author upon request.
